# Impact of orthodontic treatment on OHRQoL of adolescents: a longitudinal study

**DOI:** 10.1590/2177-6709.29.1.e2423136.oar

**Published:** 2024-02-12

**Authors:** Paula GUERINO, Fernanda Ruffo ORTIZ, Mariana MARQUEZAN, Thiago Machado ARDENGHI, Vilmar Antônio FERRAZZO

**Affiliations:** 1 Federal University of Santa Maria, Postgraduate program in Dental Sciences (Santa Maria/RS, Brazil).; 2 Federal University of Santa Maria, Department of Stomatology (Santa Maria/RS, Brazil).

**Keywords:** Orthodontics, Quality of Life, Oral Health, Adolescents, Ortodontia, Qualidade de vida, Saúde bucal, Adolescentes

## Abstract

**Objective:**

The aim of this study was to evaluate the extent to which orthodontic treatment need is perceived by the patients and by the orthodontist, as well as the possible impacts on the OHRQoL (Oral Health-Related Quality of Life) over the course of conventional orthodontic treatment in adolescent patients.

**Methods:**

The sample consisted of 55 adolescents. The perception of patients and orthodontists relative to the malocclusion was evaluated by the IOTN (Index of Orthodontic Treatment Need). The OHRQoL was evaluated by the Child-OIDP (Child-Oral Impacts on Daily Performances) questionnaire before the conventional orthodontic appliance was bonded (T0); and at the following time intervals: after one week (T1), one month (T2), three months (T3), six months (T4), and after the end of orthodontic treatment (T5).

**Results:**

Adolescents who had large orthodontic treatment needs had a poor OHRQoL, according to their self-perception (*p*=0.003) and according to the orthodontist’s perception (*p*<0.001), when compared with patients with small and moderate needs. There was statistically significant difference in the OHRQoL between the time intervals T0 and T1 (*p*=0.021), T2 and T3 (*p*<0.001), T3 and T4 (*p*=0.033), and T0 and T5 (*p*<0.002). At the end of treatment, all evaluated participants reported an improvement in OHRQoL.

**Conclusions:**

It was concluded that adolescents and orthodontists agreed with regard to the perception of orthodontic treatment need. In the first week and in the first month of orthodontic treatment, there was a negative impact on the OHRQoL. After three months, an improvement of OHRQoL was detected, which has progressed over time.

## RESUMO

**Objetivo:**

O objetivo deste estudo foi avaliar o quanto a necessidade de tratamento ortodôntico é percebida pelos pacientes e pelo ortodontista, bem como os possíveis impactos na Qualidade de Vida Relacionada à Saúde Bucal (QVRSB) ao longo do tratamento ortodôntico convencional em pacientes adolescentes.

**Métodos:**

A amostra foi composta por 55 adolescentes. A percepção dos pacientes e ortodontistas em relação à má oclusão foi avaliada pelo Índice de Necessidade de Tratamento Ortodôntico (IOTN). A QVRSB foi avaliada pelo questionário Child-OIDP (Child-Oral Impacts on Daily Performances) antes da colagem do aparelho ortodôntico convencional (T0); e nos seguintes intervalos de tempo: após uma semana (T1), um mês (T2), três meses (T3), seis meses (T4) e após o término do tratamento ortodôntico (T5).

**Resultados:**

Adolescentes que apresentavam grandes necessidades de tratamento ortodôntico apresentaram baixa QVRSB, segundo sua autopercepção (*p*=0,003) e segundo a percepção do ortodontista (*p*<0,001), quando comparados com pacientes com necessidades pequenas e moderadas . Houve diferença estatisticamente significativa na QVRSB entre os intervalos de tempo T0 e T1 (*p*=0,021), T2 e T3 (*p*<0,001), T3 e T4 (*p*=0,033) e T0 e T 5 (*p*<0,00 2 ). Ao final do tratamento, todos os participantes avaliados relataram melhora na QVRSB.

**Conclusões:**

Concluiu-se que adolescentes e ortodontistas concordaram quanto à percepção da necessidade de tratamento ortodôntico. Na primeira semana e no primeiro mês de tratamento ortodôntico, houve impacto negativo na QVRSB. Após três meses, foi detectada uma melhora na QVRSB, que progrediu ao longo do tempo.

## INTRODUCTION

Adolescence is the phase that marks the transition between childhood and adulthood, with a process of distancing from forms of behavior and typical privileges of childhood and of acquiring characteristics and skills that enable adolescents to assume the duties and social roles of adults. There are hormonal, physical, mental and social changes.^1^ The group of friends is very important at this stage, and body image is often overestimated. In this way, malocclusions can affect the adolescents’ self-esteem and even trigger bullying by members of their social community.^2^

In Orthodontics, diagnosis and treatment planning are traditionally determined by clinical and objective indicators. However, especially when treating adolescents, the patient’s chief complaint and subjective indicators related to the self-perception^3^ ^,^ ^4^ must be considered. Among the subjective measures, the Index of Orthodontic Treatment Need (IOTN) can be used in order to determine the degree of orthodontic treatment needed, taking into account the condition of malocclusion, as well as the esthetic appearance of the dentition. When this evaluation is made by the patients themselves, it’s denominated self-perception or subjective evaluation; when it’s performed by the orthodontist, it’s denominated dental or normative evaluation.^5^

During the course and at the end of the treatment, its impact also should be evaluated from the view of the patient. The change in occlusal relations resulting from orthodontic treatment, in addition to leading to esthetic, masticatory, and respiratory changes, can change the Oral Health-Related Quality of Life (OHRQoL), since the oral conditions are capable of influencing the diet, smile, speech, and socialization^6^, involving changes in relation to the psychosocial aspects of the patients’ lives.^4^ ^,^ ^7^ ^-^ ^10^

The period of adolescence is generally troubled, and OHRQoL domains related to social and emotional wellbeing issues are generally affected, thereby influencing social relations, such as displaying the teeth, laughing, and talking to other people. Furthermore, a disturbance of normal occlusion may reduce social acceptability, induce low self-esteem, and decrease OHRQoL in patients in this age range.^11^ ^,^ ^12^ Therefore, special attention must be paid to adolescent patients, who may have different esthetic postures, questions, and functional factors. Moreover, prospective studies are necessary that explore the changes in OHRQoL^8^ before and during the course of orthodontic treatment between this age group. Although there are primary studies about the impact on children and adolescents OHRQoL, its consolidation at a secondary level of scientific evidence is more focused on the times before and after orthodontic treatment, and there is no definition for the magnitude of these impacts during therapy when using appliances^13^. 

Thus, the aim of this study was to evaluate the extent to which orthodontic treatment need is perceived by the patients and by the orthodontist, as well as the possible impacts on the OHRQoL over the course of conventional orthodontic treatment in adolescent patients. The tested hypothesis was that patients would agree with professionals, and that orthodontic treatment would impact adolescents’ OHRQoL.

## MATERIAL AND METHODS

### DESIGN AND SAMPLE

This longitudinal prospective study was conducted with adolescent patients who were submitted to corrective orthodontic treatment at the Dental Clinics of the Postgraduate program in Dental Sciences of the Federal University of Santa Maria (UFSM, Santa Maria/RS, Brazil). The study was approved by the Research Ethics Committee of the UFSM (CAAE: 61189416.0.0000.5346). 

The sample size calculation was performed in the G*Power 3 software (Heinrich-Heine-Universität Düsseldorf, Düsseldorf, Nordrhein-Westfalen-NW, Germany), using the following parameters: effect size of 0.5 (moderate effect), considering a standard error of 5%, and power of 80%. Based on these parameters, a sample of 34 patients required was obtained. Calculating possible losses of 20%, the sample should be composed by 41 patients. 

The sample was selected during the years 2016 to 2018, resulting in 55 patients. The eligibility criteria were: participants had to be in the stage of permanent dentition; had to have orthodontic treatment need, irrespective of the type of malocclusion, with Class I facial profile, according to cephalometric analysis; had to be between 11 and 14 years old (due to rules of the institution’s clinic); absence of periodontal problems or caries lesions; no cognitive problems that would harm the application of the questionnaires; no corrective fixed orthodontic appliance previous use; and no need for orthodontic-surgical treatment. 

### DATA COLLECTION

The adolescents’ parents or guardians answered a questionnaire for investigating data such as: a) sex (male/female); b) skin color, which was dichotomized in “white and non-white” color; c) household income, collected in Real, representing the sum of all forms of income monthly of the family, and later dichotomized by the median (R$ 1000,00); d) parent’s schooling, collected in formal study years and categorized into 8 years, which represented formal education in Brazil; e) parent’s work; f) how many times a day the adolescents brushes their teeth, g) if the adolescent visited the dentist in the last six months, h) and whether the reason for the last dental visit was pain or routine.

The orthodontic treatment need was measured by the two components (Aesthetic Component/AC and Dental Health Component/DHC) of the IOTN.^5^ The aesthetic component is composed of 10 photographs, in decreasing order of attractiveness, and the patients were instructed to choose the image that looked most like their own dental appearance. Whereas the objective evaluation was performed in the initial plaster models by the orthodontist, using a millimeter probe, according to the dental component of the index. Classification of the orthodontic treatment needed was based on the most severe characteristic shown by each patient. To facilitate comparison between the two components, the two components of the IOTN, proposed by Lunn et al.^14^ and described in other studies^15^ ^,^ ^16^ were classified. In the above-mentioned proposal, the components are combined and divided into three levels of treatment: none/small, moderate and great. 

The OHRQoL was measured by the Child-OIDP (Child-Oral Impacts on Daily Performances) questionnaire, before the patients began with treatment (T0), one week after beginning (T1), one month (T2), three months (T3), six months after beginning the orthodontic treatment (T4), and at the end of orthodontic treatment (T5). The Child-OIDP questionnaire evaluates the impact of oral health conditions on the daily activities of adolescents. Firstly, the adolescent answers about all oral health-related problems experienced in the past three months. Afterwards, they answer questions about the severity and frequency of each impact. A score from 0 to 3 is given to rate each of the characteristics. The score of the index is a result of the multiplication of the severity and frequency of each performance. The sum is obtained for the eight performances, resulting in a number from 0 to 72, which is divided by 72 and multiplied by 100, resulting in the overall Child-OIDP score ranging from 0 to 100.^17^

All the questionnaires were applied by one single, duly trained and calibrated operator. For the assessment of malocclusion, two evaluations were made in 10 plaster models, with an interval of one week between evaluations, to determine intraexaminer (0.84) and interexaminers (0,84) agreement by the Kappa values. 

### ORTHODONTIC TREATMENT

A single operator bonded the metal fixed appliances, MBT prescription, slot 0.022 x 0.028-in (Morelli, Sorocaba/SP, Brazil), in the week after the application of the initial questionnaires. Brackets bonding began by the maxillary teeth up to the premolars, and placing the elastic separators on the maxillary and mandibular molars. In the following week, the orthodontic bands were fabricated (Morelli, Sorocaba/SP, Brazil) and placed on the maxillary and mandibular molars. In the following month, whenever possible, the mandibular brackets were bonded. The interval between return consultations was determined by the researcher in charge of the project. The duration of the orthodontic treatment varied from 2 to 5 years.

### DATA ANALYSIS

The data were analyzed in the Stata Program (StataCorp LLC, College Station, Texas-TX, USA). Firstly, the socioeconomic characteristics of the sample were demonstrated by descriptive analysis. The normality of the data was verified by Shapiro-Wilk test. Afterwards, the relations between the IOTN and OHRQoL were analyzed by Poisson adjusted regression analysis. Poisson regression allows evaluating the change in the predictor unit (IOTN) given to the referent outcome (Child-OIDP). Demographic and socioeconomic variables were included in the multivariate model to adjust the model, and to exclude possible confounding variables between OHRQoL and the need for treatment. The results were interpreted by Relative Risk (RR) and 95% confidence interval (CI). The Spearman correlation test was used to evaluate the agreement between the AC and the DHC of the IOTN. For comparison between the means of the Child-OIDP questionnaires, in the different experimental time intervals, the Wilcoxon paired statistical test was performed, due to the non-normal distribution of the data. A significance level of *p*< 0.05 was used.

## RESULTS

The sample was composed of 55 patients, with a mean age of 13 years, and the majority of patients were skin color white (85.4%). Most of the parents completed primary school education (65.4% of the mothers and 71.2% of the fathers) and worked (58.2% of the mothers and 88.9% of the fathers). Of the patients, 96% brushed their teeth at least twice a day, and this same percentage sought attendance for routine dental exams. The characteristics of the sample are described in Table 1. At the end, 23 patients finished their orthodontic treatment. Lost patients were due to city change (5 patients) and research abandonment (27 patients).

**Table 1:** Socioeconomic characteristics of the sample (n=55).

Variables	Category	N	%
Gender	Male	32	58.2
	Female	23	41.8
Skin color	White	47	85.4
	No-white	8	14.6
Household income	≤ 1 BMW	14	25.4
	> 1BMW	41	74.6
Mother’s schooling	< 8 years	19	34.6
	≥ 8 years	36	65.4
Father’s schooling	< 8 years	15	28.8
	≥ 8 years	37	71.2
Mother works	Yes	32	58.2
	No	23	41.8
Father works	Yes	48	88.9
	No	6	11.1
Brushing frequency	1x per day	2	3.6
	≥ 2x per day	53	96.4
Visit to dentist	Yes	52	94.5
(in previous 6 months)	No	3	5.5
Reason for visit	Pain	2	3.6
	Routine	53	96.4
Type of service	Private	1	1.8
	Public	54	98.2

BMW: Brazilian minimum wage (approximately US$190,00 during the data gathering).

The distribution and frequency of the items of the Child-OIDP questionnaire in the different experimental time intervals are described in Table 2, according to each item of the Child-OIDP.

**Table 2:** Frequency and distribution of items of Child-OIDP in the different experimental time intervals.

	T0	T1	T2	T3	T4	T5
Item 1 (Toothache)	14 ( 25.4)	20 ( 36.4)	13 ( 23.6)	8 ( 14.5)	3 ( 5.4)	0
Item 2 (Sensitive teeth)	18 ( 32.7)	19 ( 34.5)	14 ( 25.4)	17 ( 30.9)	10 ( 18.2)	0
Item 3 (Caries or cavity in tooth)	4 ( 7.3)	-	-	-	-	0
Item 4 (Loose primary tooth)	5 ( 9.1)	4 ( 7.3)	2 ( 3.6)	1 ( 1.8)	-	0
Item 5 (Space between teeth)	29 ( 52.7)	25 ( 45.4)	27 ( 49.1)	19 ( 34.5)	13 ( 23.6)	0
Item 6 (Broken permanent tooth)	4 ( 7.3)	4 ( 7.3)	2 ( 3.6)	2 ( 3.6)	-	0
Item 7 (Tooth color)	21 ( 38.2)	19 ( 34.5)	14 ( 25.4)	13 ( 23.6)	11 ( 20.0)	0
Item 8 (Shape or size of tooth)	17 ( 30.9)	15 ( 27.3)	13 ( 23.6)	10 ( 18.2)	7 ( 12.7)	0
Item 9 (Position of tooth)	39 ( 70.9)	35 ( 63.6)	29 ( 52.7)	28 ( 50.9)	24 ( 43.6)	0
Item 10 (Bleeding gums)	14 ( 25.4)	13 ( 23.6)	9 ( 16.4)	7 ( 12.7)	6 ( 10.9)	0
Item 11 (Swollen gum)	12 ( 21.8)	12 ( 21.8)	7 ( 12.7)	4 ( 7.3)	3 ( 5.4)	0
Item 12 (Tartar)	4 ( 7.3)	3 ( 5.4)	1 ( 1.8)	-	-	0
Item 13 (Sores in the mouth)	12 ( 21.8)	12 ( 21.8)	7 ( 12.7)	6 ( 10.9)	6 ( 10.9)	0
Item 14 Bad breath (Halitosis)	14 ( 25.4)	9 ( 16.4)	10 ( 18.2)	8 ( 14.5)	3 ( 5.4)	0
Item 15 (Deformed mouth or face)	-	-	-	-	-	0
Item 16 (Permanent tooth erupting)	14 ( 25.4)	12 ( 21.8)	8 ( 14.5)	8 ( 14.5)	4 ( 7.3)	0
Item 17 (Permanent tooth lost)	11 ( 20.0)	10 ( 18.2)	9 ( 16.4)	8 ( 14.5)	5 ( 9.1)	0
Item 18 (Others)						
Appliance	-	8 ( 14.5)	30 ( 54)	-	-	0
Separator elastic	-	23 ( 41.8)	-	-	-	0
Total	55 ( 100.0)	55 ( 100.0)	55 ( 100.0)	55 ( 100.0)	55 ( 100.0)	23 (41.81)

The mean values and standard deviation of each score of the 8 domains of the Child-OIDP are described in Table 3, in each experimental time interval. The higher mean of the overall scores were in T1 and T2, following a similar pattern across domains. At T5, all participants (n=23) did not report any discomfort in the questionnaire questions (score 0).

**Table 3:** Means and SD of overall scores and domains during orthodontic treatment in the different experimental time intervals.

	Overall scores	Domain 1 scores	Domain 2 scores	Domain 3 scores	Domain 4 scores	Domain 5 scores	Domain 6 scores	Domain 7 scores	Domain 8 scores
T0	10.95 (16.7)	1.16 (2.5)	0.62 (1.9)	0.82 (1.9)	0.13 (0.6)	0.89 (2.1)	2.45 (3.4)	0.20 (1.2)	1.61 (3.1)
T1	14.34 (21.5)	1.96 (2.8)	0.87 (2.2)	1.01 (2.3)	0.62 (1.8)	2.07 (3.5)	2.07 (3.3)	0.20 (1.2)	1.51 (3.1)
T2	14.72 (20.7)	1.89 (2.9)	1.34 (2.5)	1.58 (2.9)	0.34 (0.9)	1.82 (3.2)	2.00 (3.4)	0.16 (1.2)	1.45 (3.1)
T3	8.81 (17.8)	0.84 (2.2)	0.62 (1.9)	0.71 (2.0)	0.11 (0.4)	1.25 (2.9)	1.52 (3.1)	0.16 (1.2)	1.13 (2.7)
T4	6.08 (15.2)	0.71 (1.9)	0.42 (1.5)	0.69 (2.0)	0.11 (0.6)	0.67 (2.3)	0.94 (2.4)	0.16 (1.2)	0.67 (2.1)

SD = standard deviation. T0 = before orthodontic treatment, T1 = one week after bonding of fixed orthodontic appliance, T2 = one month after bonding of fixed orthodontic appliance, T3 = three months after bonding of fixed orthodontic appliance, T4 = six months after bonding of fixed orthodontic appliance; Domain 1 = eating, Domain 2= speaking clearly, Domain 3= cleanliness of mouth, Domain 4= sleeping, Domain 5= emotional state, Domain 6= smiling, Domain 7= studying, Domain 8= social contact

There was a moderate level of agreement between patients (AC) and orthodontists (DHC), according to the Spearman correlation (0.50, p<0.01) (results not shown in Tables).

The relations between need for orthodontic treatment and overall scores of the Child-OIDP are presented in Table 4. The patients who showed large orthodontic treatment need had poor OHRQoL (RR = 3.32; 95% CI = 2.68-4.11), when compared to small need; after adjusting by sex, age, skin color, and household income. 

**Table 4:** Relations between orthodontic treatment need (IOTN) and OHRQoL before orthodontic treatment.

IOTN	OHRQoL Mean (SD)	P
AC		0.000
Small	8.38 (11.22)	
Moderate	6.07 (8.46)	
Large	24.62 (28.43)	
DHC		0.003
Small	7.73 (10.9)	
Moderate	7.56 (8.4)	
Large	27.78 (31.0)	

SD = Standard Deviation; AC = Aesthetic Component; DHC = Dental Health Component.

The total scores of the questionnaires were compared in the different experimental time intervals, by means of the Wilcoxon paired test. There was statistically significant difference in the OHRQoL between the time intervals T0 and T1 (*p*=0.021), increase in the OHRQoL scores between T2 and T3 (*p*<0.001) and afterwards diminishing the OHRQoL scores, between T3 and T4 (*p*=0.033) and between T0 and T5 (*p*=0.002). In the analysis between the experimental times T1 and T2, there was no statistically significant difference (*p*=0.993) in the OHRQoL scores (results not shown in Tables).

## DISCUSSION

The results of this study confirmed the tested hypothesis. The findings demonstrated that the orthodontists and patients agree about orthodontic treatment needs, especially in most severe malocclusions. Moreover, there was also a significant relationship between orthodontic treatment needs and adolescents’ OHRQoL.

When the AC and DHC of IOTN were compared, it was able to observe agreement between the orthodontists and patients in the majority of cases, mainly when the orthodontic treatment need was large. Some occlusal changes are highly valued by the objective criteria of the orthodontist, however may not represent a significant esthetic compromise to the patient.^15^ The results of the present study demonstrated that the patients who had great orthodontic treatment needs had highest OHRQoL scores, when compared with the adolescents who had small or moderate orthodontic treatment needs. Similar results were obtained in a previous study.^3^ These results are probably associated with the items most affected in the evaluation of OHRQoL, related to the position of teeth and spaces between them. It can be related to the aesthetics of the patient, affecting the domain of smiling, contributing for the higher OHRQoL scores.^18^

The present study also evaluated OHRQoL throughout the course of orthodontic treatment and after the appliance removal, confirming that the beginning of orthodontic therapy had a significantly negative effect on the OHRQoL of adolescents.^19^ This was because the total OHRQoL scores increased significantly in the period of one week, after insertion of the fixed orthodontic appliance, when compared with the mean value of the OHRQoL before the orthodontic therapy. The reasons for these may be related to the painful sensitivity produced by compression of the periodontal ligament, due to the presence of the orthodontic appliance or the insertion of the elastic separators in the interproximal spaces of the molars.^20^ Moreover, during periods of adaptation to the orthodontic appliance and pain, there is a significant drop in the OHRQoL, and therefore, in our conduct, we cannot fail to provide attendance or immediate assistance.

In the evaluation of the items present in the Child-OIDP questionnaire, the item that showed the greatest change from the pre-treatment (T0) to time interval T1 was the presence of the separating elastic, followed by the fixed orthodontic appliance. The separation of teeth with elastic causes pain for 48h after insertion, tending to decrease in the next few hours.^21^ The compression forces of the orthodontic treatment led to an inflammatory response associated with the release of chemical mediators, causing an ischemic necrosis of the periodontal ligament cells, bringing pain as a consequence. Moreover, pain is a subjective sensorial experience that may be also related to psychological characteristics, such as anxiety and pain catastrophizing.^22^

In T2, the mean OHRQoL value remained stabilized, when compared with T1, but there was an increase in the frequency of the item related to the orthodontic appliance. The orthodontic appliance can cause physical and psychological changes to the patient, such as pain, discomfort, chewing difficulty, and emotional changes.^23^ In sequence, the OHRQoL scores diminished significantly in T3, in T4 and even further in T5, when compared with the mean OHRQoL value in T0. These results were in agreement with the previous study.^19^ In addition, after three and six months of orthodontic treatment beginning, the frequency of discomfort relative to the orthodontic appliance was zero, which could justify the improvement in the OHRQoL scores. This improvement in the scores could have resulted from the patients’ adaptation to the appliances and benefits that orthodontic treatment produces on the dental structures, masticatory, respiratory systems, and aesthetics.^7^ ^,^ ^19^ ^,^ ^24^

As a strong point of this study, we can mention the longitudinal follow-up of the sample patients throughout the orthodontic treatment period. The longest orthodontic treatment lasted 60 months. However, we managed to complete the orthodontic treatment in 42% of the sample. During the completion of treatments, the Covid-19 pandemic was decreed, and many patients lost follow-up due to closing of the university during the quarantine period and for personal reasons of patients and their families who gave up treatment at the institution even after resuming practical activities.

As limitations of this study, the absence of a control group could be mentioned, however, the follow-up of an untreated control is in conflict with ethical aspects, since orthodontic treatment produces benefits to the patients. Another limitation is that the IOTN is based on ten photographs, and sometimes the patient’s chief complaint may be based on another problem that is not contemplated in these static images. 

The clinical relevance of this study hinges on the discovery that adolescents perceive the severity of their malocclusion in the same way as it is evaluated by the orthodontist. While orthodontists are trained to identify the smallest changes in order to achieve normal occlusion at the end of treatment, adolescents tend to overvalue body aesthetics, including facial and dental aesthetics, so that their perceptions of the need for treatment were similar, especially in severe cases. This enhances the understanding of both adolescents and orthodontists, improving planning and patient adherence to orthodontic treatment.^23^ In addition, it is important to inform and comfort the patient that during orthodontic therapy, particularly in the beginning of it, there may be some limitations, such as pain and discomfort, and these may impact on daily performances, leading to worst OHRQoL scores. It is worth emphasizing that, for the orthodontic patient, this change in the OHRQoL scores is a temporary condition that improves over the course of the orthodontic treatment. 

## CONCLUSIONS

Based on the results, it was possible to conclude that there is a moderate agreement between the evaluations of malocclusion of adolescents by means of subjective analysis by the patient and normative analysis by the orthodontist. Also, adolescents who have great orthodontic treatment need had the poor OHRQoL, when compared with patients who have small or moderate orthodontic treatment needs. And finally, a deterioration of OHRQoL was observed in the first week and month of orthodontic treatment, showing gradual improvement after three months, six months and after the end of the treatment. 

## References

[1] Burnett S, Blakemore SJ (2009). The development of adolescent social cognition. Ann N Y Acad Sci.

[2] Gatto RCJ, Garbin AJÍ, Corrente JE, Garbin CAS (2019). The relationship between oral health-related quality of life, the need for orthodontic treatment and bullying, among Brazilian teenagers. Dental Press J Orthod.

[3] Herkrath APCQ, Vettore MV, de Queiroz AC, Alves PLN, Leite SDC, Pereira JV, Rebelo MAB, Herkrath FJ (2019). Orthodontic treatment need, self-esteem, and oral health-related quality of life among 12-yr-old schoolchildren. Eur J Oral Sci.

[4] Feu D, de Oliveira BH, de Oliveira Almeida MA, Kiyak HA, Miguel JA (2010). Oral health-related quality of life and orthodontic treatment seeking. Am J Orthod Dentofacial Orthop.

[5] Brook PH, Shaw WC (1989). The development of an index of orthodontic treatment priority. Eur J Orthod.

[6] Yusuf H, Gherunpong S, Sheiham A, Tsakos G (2006). Validation of an English version of the Child-OIDP index, an oral health-related quality of life measure for children. Health Qual Life Outcomes.

[7] Brouns VEHW, de Waal AML, Bronkhorst EM, Kuijpers-Jagtman AM, Ongkosuwito EM (2022). Oral health-related quality of life before, during, and after orthodontic-orthognathic treatment a systematic review and meta-analysis. Clin Oral Investig.

[8] Vidigal MTC, Mesquita CM, de Oliveira MN, de Andrade Vieira W, Blumenberg C, Nascimento GG, Pithon MM, Paranhos LR (2022). Impacts of using orthodontic appliances on the quality of life of children and adolescents systematic review and meta-analysis. Eur J Orthod.

[9] Liu Z, McGrath C, Hägg U (2011). Changes in oral health-related quality of life during fixed orthodontic appliance therapy an 18-month prospective longitudinal study. Am J Orthod Dentofacial Orthop.

[10] Andiappan M, Gao W, Bernabé E, Kandala NB, Donaldson AN (2014). Malocclusion, orthodontic treatment, and the Oral Health Impact Profile (OHIP-14) Systematic review and meta-analysis. Angle Orthod.

[11] Scapini A, Feldens CA, Ardenghi TM, Kramer PF (2013). Malocclusion impacts adolescents' oral health-related quality of life. Angle Orthod.

[12] World Health Organization (2017). Global Accelerated Action for the Health of Adolescents (AA-HA!): guidance to support country implementation.

[13] Pithon MM, Marañón-Vásquez GA, da Silva LP, Coqueiro RDS, Lacerda Dos Santos R, Tanaka OM, Maia LC (2022). Effect of treatment of transverse maxillary deficiency using rapid palatal expansion on oral health-related quality of life in children A randomized controlled trial. Am J Orthod Dentofacial Orthop.

[14] Lunn H, Richmond S, Mitropoulos C (1993). The use of the index of orthodontic treatment need (IOTN) as a public health tool a pilot study. Community Dent Health.

[15] Santos NR, Cabo I, Almeida F, Castro S, Ponces MJ, Lopes JD (2014). Application of the index of need for orthodontic treatment in a Portuguese orthodontic population. Rev Port Estomatol Cir Maxilofac.

[16] Cardoso CF, Drummond AF, Lages EM, Pretti H, Ferreira EF, Abreu MH (2011). The Dental Aesthetic Index and dental health component of the Index of Orthodontic Treatment Need as tools in epidemiological studies. Int J Environ Res Public Health.

[17] Castro RA, Cortes MI, Leão AT, Portela MC, Souza IP, Tsakos G, Marcenes W, Sheiham A (2008). Child-OIDP index in Brazil cross-cultural adaptation and validation. Health Qual Life Outcomes.

[18] Castro R de A, Portela MC, Leão AT, de Vasconcellos MT (2011). Oral health-related quality of life of 11- and 12-year-old public school children in Rio de Janeiro. Community Dent Oral Epidemiol.

[19] Peter E, Monisha J, Edward Benson P, Ani George S (2023). Does orthodontic treatment improve the Oral Health-Related Quality of Life when assessed using the Malocclusion Impact Questionnaire-a 3-year prospective longitudinal cohort study. Eur J Orthod.

[20] Feu D, Miguel JA, Celeste RK, Oliveira BH (2013). Effect of orthodontic treatment on oral health-related quality of life. Angle Orthod.

[21] Figueira IZ, Sousa APC, Machado AW, Habib FAL, Soares LGP, Pinheiro ALB (2019). Clinical study on the efficacy of LED phototherapy for pain control in an orthodontic procedure. Lasers Med Sci.

[22] Beck VJ, Farella M, Chandler NP, Kieser JA, Thomson WM (2014). Factors associated with pain induced by orthodontic separators. J Oral Rehabil.

[23] Baidas LF, AlJunaydil N, Demyati M, Sheryei RA (2020). Fixed orthodontic appliance impact on oral health-related quality of life during initial stages of treatment. Niger J Clin Pract.

[24] Siddiqui TA, Shaikh A, Fida M (2014). Agreement between orthodontist and patient perception using Index of Orthodontic Treatment Need. Saudi Dent J.

